# Tail Nerve Electrical Stimulation and Electro-Acupuncture Can Protect Spinal Motor Neurons and Alleviate Muscle Atrophy after Spinal Cord Transection in Rats

**DOI:** 10.1155/2017/7351238

**Published:** 2017-06-28

**Authors:** Yu-Ting Zhang, Hui Jin, Jun-Hua Wang, Lan-Yu Wen, Yang Yang, Jing-Wen Ruan, Shu-Xin Zhang, Eng-Ang Ling, Ying Ding, Yuan-Shan Zeng

**Affiliations:** ^1^Key Laboratory for Stem Cells and Tissue Engineering, Ministry of Education, Sun Yat-sen University, Guangzhou 510080, China; ^2^Department of Histology and Embryology, Zhongshan School of Medicine, Sun Yat-sen University, Guangzhou 510080, China; ^3^Department of Acupuncture, The 1st Affiliated Hospital, Sun Yat-sen University, Guangzhou, Guangdong 510080, China; ^4^Spinal Cord Society Research Center, Fort Collins, CO 80526, USA; ^5^Department of Anatomy, Yong Loo Lin School of Medicine, National University of Singapore, Singapore 117597; ^6^Institute of Spinal Cord Injury, Sun Yat-sen University, Guangzhou 510120, China; ^7^Co-Innovation Center of Neuroregeneration, Nantong University, Nantong 226001, China; ^8^Guangdong Provincial Key Laboratory of Brain Function and Disease, Zhongshan School of Medicine, Sun Yat-sen University, Guangzhou 510080, China

## Abstract

Spinal cord injury (SCI) often results in death of spinal neurons and atrophy of muscles which they govern. Thus, following SCI, reorganizing the lumbar spinal sensorimotor pathways is crucial to alleviate muscle atrophy. Tail nerve electrical stimulation (TANES) has been shown to activate the central pattern generator (CPG) and improve the locomotion recovery of spinal contused rats. Electroacupuncture (EA) is a traditional Chinese medical practice which has been proven to have a neural protective effect. Here, we examined the effects of TANES and EA on lumbar motor neurons and hindlimb muscle in spinal transected rats, respectively. From the third day postsurgery, rats in the TANES group were treated 5 times a week and those in the EA group were treated once every other day. Four weeks later, both TANES and EA showed a significant impact in promoting survival of lumbar motor neurons and expression of choline acetyltransferase (ChAT) and ameliorating atrophy of hindlimb muscle after SCI. Meanwhile, the expression of neurotrophin-3 (NT-3) in the same spinal cord segment was significantly increased. These findings suggest that TANES and EA can augment the expression of NT-3 in the lumbar spinal cord that appears to protect the motor neurons as well as alleviate muscle atrophy.

## 1. Introduction

Spinal cord injury (SCI) is a worldwide problem and causes immense suffering and burden to the patient. Traumatic injuries to the spinal cord disrupt the transmission of both ascending sensory projections and descending projections to motor neurons, leading to permanent sensory and motor functional deficits [1]. Although the motor neurons in the ventral horn of the spinal cord below the lesion site retain their connection to the target muscles, the loss of ascending and descending stimulation makes the spinal motor neurons shrink and die, resulting in atrophy of the innervated muscles. Natural neural regenerative response following SCI is very little, if any; meanwhile, however, the muscle atrophy continues and the change is remarkable within a short time [2, 3]. There are two main strategies to minimize the muscle hypotrophy after SCI, and this includes using physical exercises to drive the disused hindlimbs [4, 5] and giving a localized electrical stimulation to the atrophic muscles [6, 7]. It was thought that external intervention like locomotor training or functional electrical stimulation could provide sensory inputs and effectively reactivate the lumbar motor circuit, giving a rise to the neuromuscular activity.

The tail is an important organ of the rat with various functions involved in sensation, temperature control, balance, and movement [8]. Tail nerve electrical stimulation (TANES) for SCI therapy was first put forward and used by Zhang et al. They found that electrical stimulation on the tail of a rat was conducive to locomotor recovery in a model of rat spinal cord contused, and this was ascribed to the activation of central pattern generator (CPG) through the tail nerve [9].

Electroacupuncture (EA) is a traditional Chinese medical method that is widely used for various research studies and clinical applications [10–12]. EA has long been used in SCI therapy and, in this connection, it has been shown to inhibit inflammation, promote neurotrophic factor secretion, reduce secondary injury, and so forth [13–15]. We also reported previously that EA stimulation on the Governor Vessel could promote the regeneration of nerve fibers in the injury site of the spinal cord [16, 17]. Although the work theory of EA stimulation remained uncertain, with the anatomical investigation showing that acupoints tend to be adjacent to nerve fibers [18] and the experimental evidence that complete denervation totally suppresses the effect of the points [19, 20], the effect of EA may involve convergence of nerve impulses on the primary afferent sensory fibers into the spinal cord.

Neurotrophic factors (NTFs) are a family of proteins that are essential to the development, survival, and function of neurons [21–23]. The protein neurotrophin-3 (NT-3) is a member of the neurotrophin family and has been demonstrated in experimental animal models to have an important role in neuroprotection and axonal regeneration, yet its localized and sustained delivery remains challenging [24, 25]. Giving EA treatment may be a potential strategy to cope with this problem. Previous research found that EA on Governor Vessel has a beneficial effect on the improvement of endogenous NT-3 in the injury area of the spinal cord at different time points after SCI [26–28]. However, it remains to be ascertained whether EA would influence the NT-3 secretion at the spinal lumber segment and whether TANES could affect the expression of NT-3 as well.

As mentioned above, the normal lumbar spinal neurons are implicated after SCI, which directly affects the hindlimb muscles. To patients with severe SCI who often are not able to perform physical exercise, electrical stimulation may be an option for rewiring the lumbar spinal neural plasticity to relieve muscle atrophy. In addition to electrical stimulation, TANES or EA might offer activity-dependent plasticity as well. However, the study of TANES was mainly concerned with the locomotor behavior change for contused spinal cord of rat, while the spinal pathophysiological changes had remained unexplored [9, 29, 30]. In EA study, the investigation was extended to the behavioral reaction and focused on structural change in the injury site of the spinal cord [26, 28]. It was reported that acupuncture on the hindlimbs ameliorated skeletal muscle atrophy induced by hindlimb suspension in mice or denervation in rats [31, 32]. It remains to be ascertained whether TANES and EA could offer a protective effect of lumbar spinal motor neurons and alleviate muscle atrophy of rat with SCI. The present study was designed and sought to explore if TANES or EA stimulation to rats with a complete transection of the spinal cord could promote NT-3 secretion, protect lumbar spinal motor neurons, and reduce muscle atrophy of hindlimbs.

## 2. Experimental Procedures

### 2.1. Animals and Experimental Groups

Adult female Sprague-Dawley (SD) rats (*n* = 72, 220–250 g, supplied by the Experimental Animal Center of Sun Yat-sen University) were randomly assigned into four groups: (1) spinal cord injury group (the SCI group), (2) tail nerve electrical stimulation treatment group (the TANES group), (3) electroacupuncture treatment group (the EA group), and (4) sham operation group (the Sham group). There were 18 rats in each group; half of them were used for morphological analysis and the remainder were used for Western blot analysis.

### 2.2. Spinal Cord Surgery

The spinal transection surgery was previously described [28]. Briefly, animals were anesthetized with 1% pentobarbital sodium (40 mg/kg, i.p.). Under the sterile condition, a laminectomy was carried out at the T9 vertebral level to expose the T10 spinal segment. The dura was excised, and the T10 spinal segment was transected completely with an ophthalmic scissors and with no tissue removed. The rats for the Sham group were subjected to vertebral lamina and dura resection only without spinal cord transection. After adequate hemostasis, the overlying muscles, fascia and skin incision were closed in layers using sutures. In postsurgery care, manual urination was given three times daily, and an intramuscular injection of penicillin (160,000 U/ml/d) was administered to prevent infection for all animals.

### 2.3. TANES or EA Treatment

**Figure 1:**
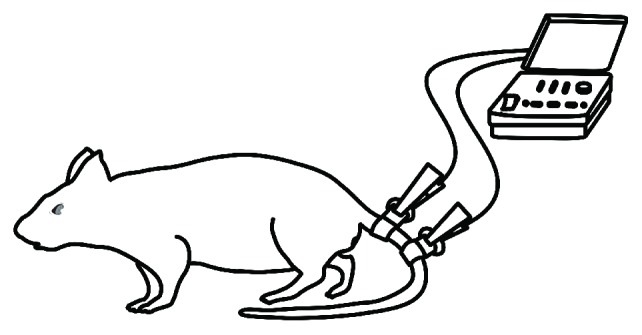
A schematic diagram shows the method of the tail nerve electrical stimulation conducted by computer control middle frequency instrument.

TANES treatment was carried out according to the protocol reported by Dr. Shu-Xin Zhang with some modification [28]. The therapy was started on the third day postsurgery and given 5 times a week for 4 weeks. Without anesthesia, the rat was kept in an open field (a square box with side length of one meter) to allow its free movement and two electrodes were placed adequately apart (to avoid a short circuit) on the base of the tail (Figure 1). The two electrodes were connected to a physical therapy instrument (Type J18A1 computer control middle frequency instrument, Quan-Ri-Kang Company, China; it has demonstrated no risk and no side effects in applications in both clinic and home). The instrument possesses a middle frequency carrier wave of 2.5–8.0 kHz and low adjustable frequency 1–150 Hz with maximal output current 100 mA. The strength of stimulation was adjusted to 20 mA at a frequency of 4 kHz to induce a slight vibration of the tail or twitch of the hindlimbs, and the treatment lasted for 20 min every time for each rat.

**Figure 2:**
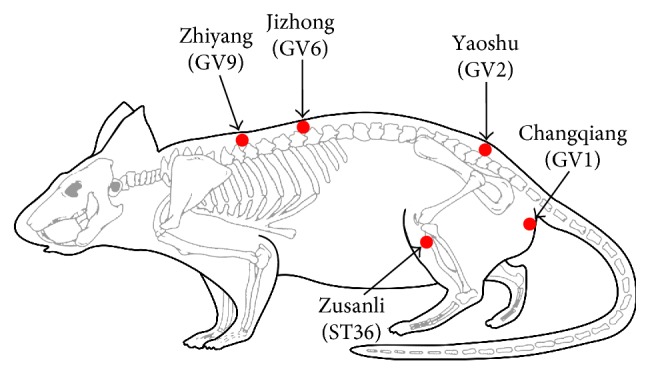
A schematic diagram indicates location of selected electroacupuncture acupoints in rat. Arrows show the four Governor Vessel acupoints: Jizhong (GV6), Zhiyang (GV9), Yaoshu (GV2), and Changqiang (GV1) and the bilateral Zusanli (ST36) in hindlimbs.

EA stimulation was administrated at three pairs of acupuncture points, including two pairs of Governor Vessel acupoints: (1) GV9 (*Zhiyang*) and GV6 (*Jizhong*) points; (2) GV2 (*Yaoshu*) and GV1 (*Changqiang*) points); and two ST36 (*Zusanli*) points in the hindlimbs (Figure 2). The location of Governor Vessel acupoints followed that previously described [33]. The ST36 is 5 mm beneath the capitulum fibulae and is located laterally and posterior to the knee joint [34, 35]. The rats were kept in a specially designed restraint equipment without anesthesia such that they remained in a recumbent position during the EA treatment. Stainless acupuncture needles (0.30 mm in diameter, 50 mm in length; Jiangsu Medical Instruments Inc., China) were inserted at a depth of 5 mm [16] into the four Governor Vessel acupoints, and needles (0.25 mm in diameter, 13 mm in length) were inserted at a depth of 3 mm [36] into the bilateral ST36 points. The three pairs of needles were connected to the output terminals of an EA apparatus (model number G 6805-2, Shanghai Medical Electronic Apparatus Company, China), and EA was applied using alternating strings of dense-sparse waves at alternating frequencies (60 Hz for 1.05 s and 2 Hz for 2.85 s, pulse width 0.5 ms). The intensity was adjusted to induce slight twitching of the hindlimbs (≤1 mA), and each treatment lasted 20 min. EA treatment was given once every other day for 4 weeks and started on the third day postsurgery.

### 2.4. Morphological Quantification

All rats were sacrificed 30 days after surgery. They were deeply anesthetized with 1% pentobarbital sodium (50 mg/kg, i.p.) and weighed and then perfused transcardially with 4% paraformaldehyde. The spinal cord and hindlimbs were dissected and removed from each animal. The spinal cord was postfixed overnight in cold 4% paraformaldehyde and then transferred to 30% sucrose/phosphate-buffered saline (PBS) for 3 days. To analyze the survival of injured motor neurons innervating the hindlimbs, the lumbar segments (L3 and L5) of the postfixed spinal cord were dissected and sectioned transversely at 20 *μ*m thickness with a cryostat. Every 5th section of the spinal cord segment was collected. A total of 10 sections from each segment per rat were used for neuronal counts after neutral red staining. The number of motor neurons with intensely stained Nissl substance in the cytoplasm and well-delineated nucleus in the ninth lamina of the right and left ventral horns for each section was counted under a light microscope at the magnification of 200x [37]. The number of motor neurons in two sides of the ventral horns of each section was pooled to yield a total number of cells per section. Finally, the total number of neurons of 10 sections derived from each rat was presented for statistical analysis.

### 2.5. Western Blot Analysis

Western blot analysis was used to detect the expression levels of choline acetyltransferase (ChAT) and NT-3 proteins in the lumbar spinal cord at 30 days after surgery. Briefly, the L5 spinal cord segment (2.5 mm in length) was dissected after intracardial perfusion of the rat with PB under the anesthesia. The segment was lysed in RIPA buffer (Sigma-Aldrich) containing 2% protease inhibitor cocktails (Roche, Germany). After centrifuged at 12,000 rpm for 20 min at 4°C, the supernatant was collected and stored in −80°C for Western blots. The protein concentrations were determined using a BCA Protein Assay kit (Thermo Scientific™ Pierce). Equal amounts of protein (40 *μ*g) were resolved on 12% SDS-polyacrylamide gel electrophoresis and transferred to PVDF membranes (Millipore, Massachusetts, USA). The membranes were rinsed in TBST, blocked at room temperature for 1 h in BSA Blocking Buffer, and incubated with the following primary antibodies at 4°C overnight: rabbit anti-ChAT (1 : 1000, Proteintech Group, Inc.), rabbit anti-NT-3 (1 : 1000, Santa Cruz Biotechnology, Santa Cruz, USA), and mouse anti-*β*-Tubulin (1 : 2000, Proteintech Group, Inc.). After rinsing with TBST, the membranes were incubated with the HRP-conjugated goat anti-mouse and rabbit IgG (1 : 5000, Invitrogen Life Technologies, Carlsbad, CA, USA) for 1 h at room temperature. To visualize the immunoreactive proteins, the enhanced ECL kit (CWBIO, Beijing, China) was used following the manufacturer's instructions. Quantitative densitometry of captured images was analyzed with ImageJ (NIH, USA).

### 2.6. Immunofluorescence

The sections of the spinal cord were rinsed with 0.01 M phosphate-buffered saline (PBS) three times, blocked with 10% goat serum for 30 min, and incubated with primary antibodies mixed in 0.3% Triton X-100 overnight at 4°C. After rinsing with PBS, the sections were incubated with the secondary antibodies and examined under a fluorescence microscope (Olympus BX63, Tokyo, Japan). The antibodies used are as follows: rabbit anti-NT-3 (1 : 200, Abcam, London, UK), mouse anti-NeuN (1 : 1000, Sigma-Aldrich, St. Louis, USA), rabbit anti-ChAT (1 : 50, Proteintech Group Inc., Chicago, IL, USA), Alexa Fluor 488 goat anti-rabbit antibody (1 : 500, Cell Signaling Technology Inc., Danvers, MA, USA), Alexa Fluor 555 goat anti-mouse antibody (1 : 1000, Cell Signaling Technology Inc.).

### 2.7. Muscle Atrophy Analysis

Before postfixation, the gastrocnemius muscles were weighed in a precise weighing scale (0.01 g) after careful dissection of associated tendons and fascia. To reduce deviation, the smaller mass gastrocnemius muscle of each rat was chosen for mass and muscle fiber cross-sectional area analysis. The midbelly of muscles were dehydrated, embedded in paraffin, and sectioned transversely at 4 *μ*m thickness with a paraffin microtome (Leica RM 2235, Germany). One section of every 100 *μ*m of the selected muscle segment and a total 5 sections of each rat were collected for haematoxylin and eosin (HE) staining. Images of random fields of each muscle section were captured under a bright-field microscope (Leica DMIRB) at a typical magnification of 200x. Muscle fiber area in the gastrocnemius was determined using the ImageJ software (NIH, USA).

### 2.8. Statistical Analysis

Data were analyzed using SPSS 13.0 software. All values were expressed as mean ± standard deviation (SD). Comparisons between groups were evaluated by one-way ANOVA and post hoc statistical test (LSD). The statistically significant difference between samples was accepted if *p* < 0.05.

## 3. Results

### 3.1. TANES or EA Treatment Can Protect the Motor Neurons

**Figure 3:**
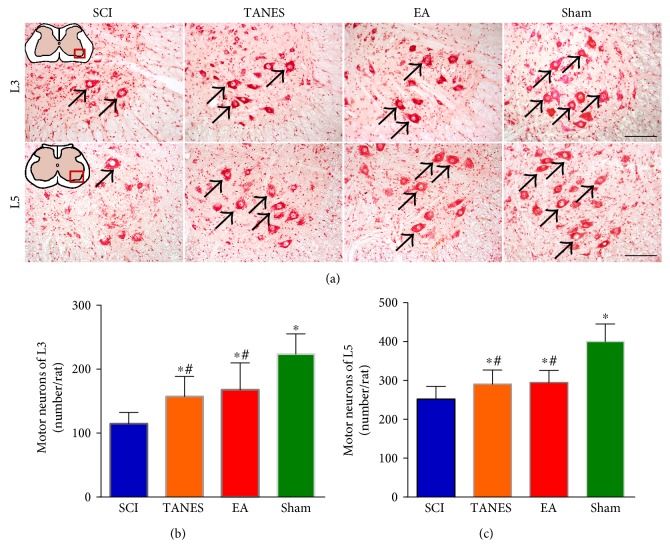
Tail nerve electrical stimulation and electroacupuncture treatment can protect the motor neurons in the ventral horn of the lumbar spinal cord. (a) Representative motor neurons stained with neutral red are located in the lumbar spinal cord (L3 and L5). (b, c) Quantification of the number of motor neurons survived at per rat in L3 (b) and L5 (c) of the SCI, TANES, EA, and Sham groups. Data are presented as mean ± SD. ^∗^*p* < 0.05, as compared with the SCI group; ^#^*p* < 0.05, as compared with the Sham group. Scale bars = 100 *μ*m in (a).

The motor neurons governing the hindlimb muscles were mainly located in the lumbar spinal cord. To determine the neuroprotection of TANES or EA treatment, the survival of motor neurons (L3 and L5 segments) in the transected spinal cord was evaluated after the neutral red staining. The motor neurons in the ninth lamina were readily recognized by their large cell bodies (soma) and thick easily stained Nissl substance. An ANOVA showed that the spinal cord injury had an obvious effect on the survival of motor neurons, *F*_(3, 32)_ = 17.8, *p* < 0.05 for L3 and *F*_(3, 32)_ = 27.1, *p* < 0.05 for L5. In the TANES and EA groups, the number of surviving motor neurons with relatively normal morphological features (Figures 3(a), 3(b), and 3(c)) was significantly higher than that in the SCI group (*p* < 0.05 in L3; *p* < 0.05 in L5); it was, however, lower when compared with the Sham group (*p* < 0.05). There was no significant difference in the number of motor neurons between the TANES and EA treatment groups (*p* > 0.05).

### 3.2. Spinal Neurons Exhibited More Intense ChAT Expression after TANES or EA Treatment

**Figure 4:**
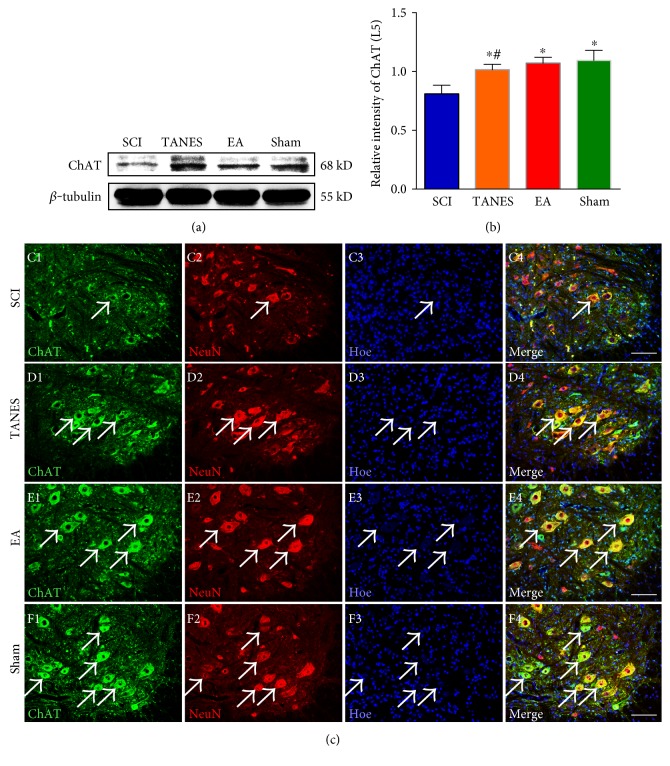
Spinal neurons expressed more excitatory neurotransmitter synthetase—ChAT in L5 spinal cord after TANES or EA treatment. (a) The expression of ChAT in L5 spinal cord is detected with Western blot among the four groups. (b) Quantitative analysis of data in (a). Data are presented as mean ± SD. ^∗^*p* < 0.05, as compared with the SCI group; ^#^*p* < 0.05, as compared with the Sham group. (c, d, e, f) ChAT (green) and NeuN (red) double-label immunofluorescence staining shows the expression of ChAT in L5 motor neurons from the SCI, TANES, EA, and Sham groups. Scale bars = 100 *μ*m in (c, d, e, f).

The innervation of the hindlimb muscles by the lumbar motor neurons is known to be mediated by the excitatory neurotransmitter acetylcholine. In view of this, the expression of ChAT (a transferase enzyme responsible for acetylcholine synthesis) in L5 segment of the spinal cord was analyzed by Western blot. The statistical result showed a significant decrease in ChAT expression after SCI, *F*_(3, 32)_ = 35.1, *p* < 0.05. Post hoc comparisons revealed that the SCI group differed from the Sham group (Figures 4(a) and 4(b), *p* < 0.05). In the TANES group or EA group, ChAT expression in L5 spinal cord was partially restored and was significantly (*p* < 0.05) higher than the SCI group; however, there was no significant difference in ChAT expression between the TANES and EA groups (*p* > 0.05). Immunofluorescence staining showed that ChAT was widely expressed in the somata and processes (mainly identified as dendrites) of motor neurons (Figures 4(c), 4(d), 4(e), and 4(f)). Consistent with the semiquantitative numerical assessment, colocalization study of ChAT and NeuN showed an intense ChAT immunoreactivity in the motor neurons in the Sham group (Figure 4(f)). In the SCI group, ChAT immunoreactivity in the motor neurons was markedly reduced so that it was only weakly detected (Figure 4(c)); however, it was increased after TANES or EA treatment (Figures 4(d) and 4(e)). The results suggest that either one of the two treatments can prevent muscle atrophy and death of motor neurons. Additionally, both can enhance the synthesis of neurotransmitter through ChAT.

### 3.3. NT-3 Expression Level Was Enhanced in Lumbar Spinal Cord after TANES or EA Treatment

**Figure 5:**
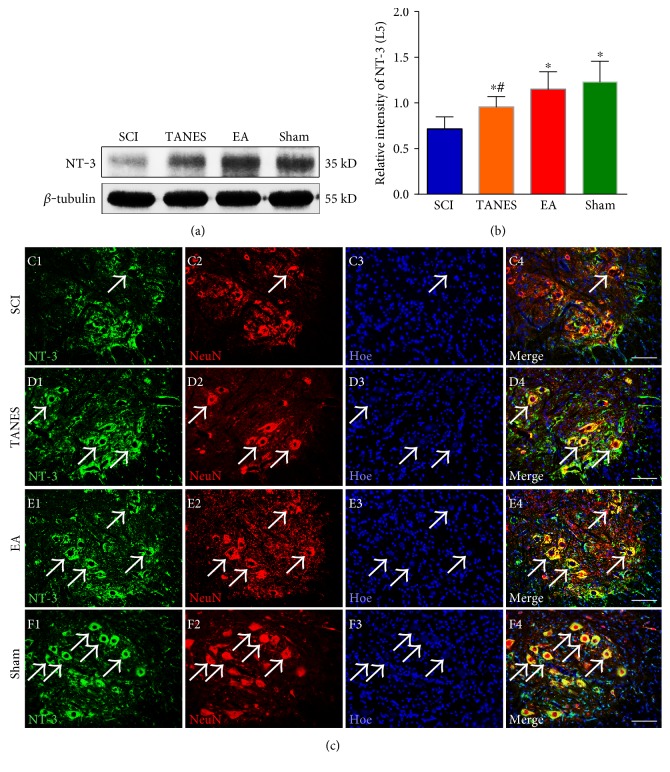
NT-3 expression was enhanced in L5 spinal cord segment after TANES or EA treatment. (a) The expression of NT-3 in L5 spinal cord is detected with Western blot among the four groups. (b) Quantitative analysis of data in (a). Data are presented as mean ± SD. ^∗^*p* < 0.05, as compared with the SCI group; ^#^*p* < 0.05, as compared with the Sham group. (c, d, e, f) NT-3 (green) and NeuN (red) double-label immunofluorescence staining displays the expression of NT-3 in L5 spinal cord among the four groups. Scale bars =100 *μ*m in (c, d, e, f).

Neurotrophins are well known for their beneficial effects on neuroprotection and neural plasticity. NT-3 is expressed in numerous cell types in the spinal cord, including motor neurons, interneurons, astrocytes, and oligodendrocytes, and plays an important role on withstanding injury-induced neuronal death [38]. We used Western blot analysis to determine the expression of NT-3 semiquantitatively for assessing the progression of SCI and the protective effects of TANES and EA. The level of NT-3 in L5 segment of the spinal cord was reduced after SCI, *F*_(3, 32)_ = 15.0, *p* < 0.05. Post hoc comparisons showed that the expression of NT-3 in the SCI group was significantly lower than that of the Sham group (*p* < 0.05, Figures 5(a) and 5(b)); however, the expression level of NT-3 was significantly enhanced in the TANES and EA groups when compared with that in the SCI group (*p* < 0.05). In comparison with the Sham group, NT-3 expression was significantly lower in the TANES group (*p* < 0.05). NT-3 expression in the EA group, however, was not significantly different from the Sham group (*p* > 0.05). There was no statistical significance in NT-3 expression between the TANES and EA groups (*p* > 0.05). Similarly, in the NT-3 immunoreactivity observation, the green fluorescence staining in Figure 5 showed a robust expression of NT-3 in L5 neural cells in the TANES, EA, and Sham groups relative to the SCI group. Double-immunofluorescence staining of NT-3 and NeuN showed the expression of NT-3 in the neurons of L5 spinal cord segment (Figures 5(c), 5(d), 5(e), and 5(f)). NeuN-positive neurons with their intense fluorescence appeared to overlap or showed total coincident with NT-3 fluorescence in the Sham group. In the SCI group, however, NT-3 was confined to only partial areas in the cell body of NeuN-positive neurons (Figure 5(c), arrow); on closer examination, some NeuN-positive cells were devoid of NT-3 labeling (Figure 5(c)).

### 3.4. Skeletal Muscle Atrophy Is Alleviated by TANES or EA Treatment

**Figure 6:**
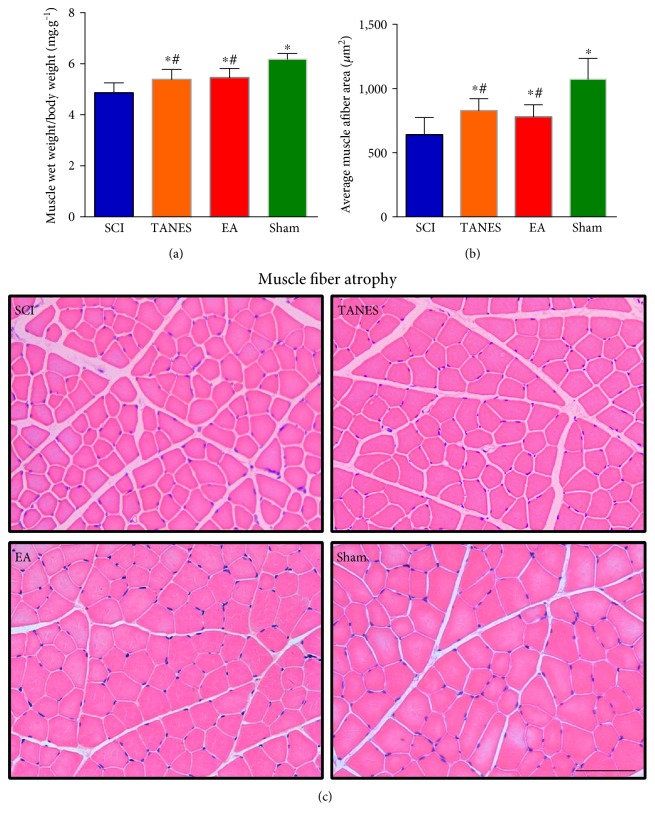
Skeletal muscle atrophy following spinal cord injury was alleviated by tail nerve electrical stimulation or electroacupuncture treatment. (a) Showing the change of wet weight of gastrocnemius muscle relative to body weight. (b) Quantification of cross-sectional area (*μ*m^2^) of gastrocnemius muscle fibers. (c) Representative muscle fibers stained by hematoxylin and eosin are displayed in the SCI, TANES, EA, and Sham groups, respectively. Data are presented as mean ± SD. ^∗^*p* < 0.05, as compared with the SCI group; ^#^*p* < 0.05, as compared with the Sham group. Scale bars = 100 *μ*m in (c).

To assess the histological alterations of hindlimb muscle 28 days after SCI, the change of wet weight and muscle fiber cross-sectional area of gastrocnemius muscle was analyzed. For muscle mass analysis, the relative muscle wet mass (muscle weight/body weight) of each rat was used to eliminate the individual variation in body weight. A complete transection of the spinal cord could cause severe skeletal muscle atrophy. In accord with the decrease of the number of surviving motor neurons, SCI did have a significant effect on muscle weight, *F*_(3, 32)_ = 21.9, *p* < 0.05 and cross-sectional area of muscle fibers, *F*_(3, 32)_ = 19.0, *p* < 0.05. The statistical analysis showed that the wet weight and cross-sectional area of muscle fibers in the gastrocnemius muscle were significantly smaller in the SCI group than those in the Sham group (*p* < 0.05, Figures 6(a) and 6(b)). In typical HE staining images, there was obvious change, that is, reduction in the diameter of muscle fibers (Figure 6(c)). It was also evident that atrophy of muscle fibers could be alleviated partially with TANES or EA treatment, as compared with the SCI group (*p* < 0.05, Figures 6(a), 6(b), and 6(c)). However, the muscle conditions in terms of muscle weight and muscle fiber diameter of the rat treated by TANES or EA treatment were not fully restored to the normal level when compared with the Sham group (*p* < 0.05, Figures 6(a), 6(b), and 6(c)). Furthermore, there was no significant difference in the muscle conditions between the TANES and EA groups (*p* > 0.05, Figures 6(a), 6(b), and 6(c)).

## 4. Discussion

The present study investigated whether TANES or EA treatment could protect motor neurons of the lumbar spinal cord controlling the muscles of the hindlimbs and whether they would alleviate the skeletal muscle atrophy after complete SCI. Concurrent to this, we also compared the therapeutic effects between TANES and EA treatment. We showed here that TANES or EA treatment has a protective effect on the lumbar spinal motor neurons and prevents at least to a certain extent hindlimb muscle atrophy caused by SCI. Interestingly, experimental evidence gained from this study indicates that there was no significant difference in the therapeutic effect of TANES and EA.

Thoracic spinal cord transection causes the interruption of brain innervation to the lumbar segments which results in the neuronal death at the lumbar spinal cord. This ultimately leads to atrophy of the innervated muscles. Giving exterior intervention to reactivate the lumbar segments may be a feasible treatment strategy for relieving the muscle atrophy [39]. There is available evidence demonstrating the beneficial effect of electrical stimulation (ES) on muscle atrophy treatment [7, 32, 40]. It is believed that electrical stimulation can reactivate the spinal motor neurons by offering sensory afferent stimulation, and this would then accelerate the neuromuscular performance recovery to reduce muscle atrophy and fibrosis formation [41, 42]. TANES is a mode of electrical stimulation applied at the tail. The rat tail is very sensitive and has many functions with profuse sensory and motor fibers derived from the spinal cord [43, 44]. It has been reported that passive movement of the tail can strongly influence the activity of neurons in the spinal cord [45]. Grau et al. found that controllable electrical stimulation applied to the rat tail of transected spinal cord can offer adaptive plasticity within lumbar interneuronal populations of putative central pattern generators (CPG) to promote spinal learning [46, 47]. The work from Zhang et al. also confirmed that TANES can activate the CPG in the lumbar spinal cord to promote the locomotor recovery of contused spinal cord of rat [9, 30]. Here, we demonstrated for the first time the therapeutic efficacy of TANES on spinal neuron protection and atrophy muscle improvement in an animal model of complete SCI.

EA is more widely studied and used in experimental and clinical research relative to TANES. The application of acupuncture for treatment of SCI has shown promising results in inflammation inhibition and beneficial factors secretion [48, 49]. Improving the microenvironment after injury is very helpful for survival of the spinal cord neurons and functional reconstruction [50]. There is also experimental evidence supporting that acupuncture therapy in SCI can reduce apoptosis of both neurons and oligodendrocytes in the injury area [51]. Besides, when combined with cell transplantation, EA can enhance the survival and differentiation of transplanted cells, promote the axonal regeneration, and reconnection with the host neurons [28]. The present results have further extended that EA is beneficial for survival of lumbar motor neurons in SCI and that it improves atrophy of muscles affected by the cord injury.

It is unequivocal from the present results that TANES or EA treatment could protect motor neurons of the lumbar spinal cord controlling the hindlimbs and alleviate the atrophy of skeletal muscles after complete SCI at the thoracic level. The issue arose from this would be the underlying mechanism of the effectiveness of TANES or EA treatment. It is known that neurotransmitters are the function carriers for motor neurons to the target muscle. The hindlimbs are dominated by sciatic nerves. Anatomically, 60%–70% spinal fibers that make up the sciatic nerve are derived from L5 segment in Sprague-Dawley rats [52, 53]. Furthermore, the study of nerve degeneration caused by spinal nerve ligation indicated that L5 is the primary contributor to the sural branches of the sciatic nerve [54], making L5 as the detection target of ChAT which is a transferase enzyme responsible for the synthesis of the excitatory neurotransmitter acetylcholine. As expected, the expression levels of ChAT in the TANES and EA groups were higher than those of the Sham group. This suggests that the reduced muscle atrophy after SCI coupled with TANES or EA treatment may involve ChAT expression level in the lumbar spinal cord. In addition, the local delivery of neurotrophins can counteract pathological events and induce a regenerative response in either acute or chronic spinal cord injury [55–57]. It is well documented that NT-3 is an important regulator for the survival, differentiation, and function of neurons in the central nervous system [58, 59]. Our previous studies suggested that EA on the Governor Vessel could promote the expression of NT-3 in the injured area of the spinal cord, which was not only good to the differentiation and integration of transplanted cells but also well to the survival of host neurons and their axon regeneration [28, 60]. In this study, we confirm that TANES or EA stimulation can enhance the expression of NT-3 in the lumbar spinal cord in which there are motor neurons innervating the hindlimb musculature. NT-3 is important for sensory afferent projections to spinal motor neurons and can modulate the connectivity between them [61–63]. In consideration of the above, it is suggested that the effect of TANES or EA treatment in increasing the survival of motor neurons as well as alleviating the hindlimb muscle atrophy may be associated with NT-3 and ChAT. Interestingly, TANES and EA can induce a similar effect on atrophy muscle and the related spinal neurobiological change. This is consistent with the view that the artificial afferent input is beneficial for spinal plasticity [64]. Our study might offer the experimental evidence to explain the effectiveness of EA intervention treatment on the recovery of nerve injury diseases in clinical practice.

## 5. Conclusion

The present results have demonstrated that, following a complete transection of the lower thoracic spinal cord segment in the rat, TANES or EA treatment can increase the expression of NT-3 and ChAT localized specifically in the lumbar motor neurons in the ventral horns. More importantly, either one of the treatments can prevent the secondary injury to the spinal motor neurons. Equally important is the fact that the treatment can alleviate the muscle atrophy in the hindlimb. Arising from this, it is suggested that TANES or EA treatment in the spinal cord injury should be further explored as a potential strategy for treating complex neurological maladies.
